# Weathering the Storm: How Age and Biologics Influence the COVID-19 Cytokine Surge

**DOI:** 10.3390/pathogens14040346

**Published:** 2025-04-03

**Authors:** Corine Astroth, Karishma S. Shah, Sudhanshu Agrawal, Anshu Agrawal

**Affiliations:** Division of Basic and Clinical Immunology, Department of Medicine, University of California Irvine, Irvine, CA 92697, USA; castroth@hs.uci.edu (C.A.); karisss1@hs.uci.edu (K.S.S.); sagrawal@uci.edu (S.A.)

**Keywords:** COVID-19, elderly, cytokine storm, biologics, therapeutics

## Abstract

SARS-CoV-2, first identified in December 2019, caused a global pandemic, resulting in over 6.8 million deaths by March 2023. The elderly, or individuals over 65, accounted for the majority of COVID-19 deaths, with 81% of fatalities in the US in 2020 occurring in this group. Beyond mortality, aging populations are also at higher risk of long-term cardiovascular complications and acute respiratory distress syndrome (ARDS). Although these outcomes may be influenced by comorbidities common in the elderly, age has been found to be a standalone risk factor for severe COVID-19 infection. Therefore, investigating age-related factors in COVID-19 outcomes is crucial in protecting this vulnerable group. Of particular interest is the cytokine storm phenomenon, an excessive inflammatory response that contributes to severe COVID-19 symptoms, including ARDS and cardiovascular damage. Elevated levels of multiple cytokines are common in severe cases of COVID-19. We propose that changes that occur to cytokine profiles as we age may contribute to these aberrant inflammatory responses. This review specifically explored the interleukin class cytokines IL-1, IL-6, IL-17, and IL-23 and considered the potential of biologics targeting these cytokines to alleviate severe outcomes in both COVID-19 and aging individuals.

## 1. Introduction

In 2019, the novel virus SARS-CoV2 was first detected. It would go on to ravage the population, leading to over 6.8 million deaths worldwide between its conception in December 2019 and March 2023. These deaths were not equally distributed, however, with the elderly population, or those over the age of 65, making up the majority of the death toll [1]. In the US in 2020, 350,381 people died of COVID-19, with 81% of those people being elderly [2]. Beyond mortality, the clinical consequences for aging populations are also of concern. Among the very elderly COVID-19 patients, 26% of them had long-term cardiovascular complications including atrial fibrillation, pulmonary thromboembolism, deep vein thrombosis, myocardial ischemia, and myocarditis [3]. Additionally, the elderly were more likely to develop acute respiratory distress syndrome (ARDS), which was not only a risk factor for mortality but also comes with risk for numerous long-term complications such as cognitive impairment and physical weakness [4,5]. Finally, the elderly population may experience long COVID, a syndrome in which symptoms attributed to COVID-19 infection persist for more than 12 weeks [6]. Despite age not being associated with increased risk for long covid symptoms, it still presents a problem for the elderly population, and additional reporting may suffer numerous biases, including survivor bias, given the high rates of death in the elderly population [7]. Although some of these differences in morbidity and mortality may be attributed to comorbidities common in the aging population, the observed differences are only part of the picture given that age is a standalone risk factor for severe infection [8]. Thus, the impact of age on COVID-19 outcomes warrants further investigation to better protect the vulnerable aging population.

Cytokines are of particular interest given the role of cytokine storm in severe COVID-19 infection, which results in death or disability. Cytokine storm is a phenomenon in which the body releases cytokines at an increased rate over a short period of time, resulting in a severe immune reaction. One of the main roles of the immune system is to identify possible harmful foreign pathogens and stage an inflammatory response that clears these agents from the body. Cytokines assist in this process by helping immune cells properly communicate with one another. However, if this process goes awry, the inflammatory response can be great enough to cause extensive damage to native cells and tissues as well. This phenomenon can be seen in a variety of infections, autoimmune conditions, and hematologic malignancies that, if severe, may even cause end organ damage. In each of these cases, the ultimate problem is an excessive inflammatory response; however, the mechanism may differ depending on the offending agent. Generally, it is thought that the process emerges due to the presence of a positive feedback loop in which cytokines activate inflammatory cell death pathways, leading to cell death, and then are released at an increased rate as cells die [9,10]. In infections, the immune system recognition of pathogen-associated molecular patterns (PAMPs) or damage-associated molecular patterns (DAMPs) leads to activation of NF-kB, MAPK, and interferon regulatory factor 3 (IF3)-IF7, subsequently leading to the transcription of pro-inflammatory cytokine genes or through inflammasome sensors that form inflammasome complexes containing caspases that cleave cytokines and create pores in cells, eventually releasing cytokines into the environment. On the other hand, in autoimmune conditions, the immune system derangement may begin at the genetic level such as in primary Hemophagocytic Lymphohistiocytosis (HLH) where mutations impacting T cell and NK cell activity leads to excessive pro-inflammatory cytokine production [11].

In COVID-19, patients that suffered from severe infection were found to have higher levels of chemokines including CXCL-10, CCL-2, CCL-4, cytokines such as soluble IL-2R, IFN-γ, IL-6, IL-10, and TNF-α, as well as C-reactive protein (CRP) [12,13], with reports of up to 92.3% of individuals with cytokine storm requiring treatment in the intensive care unit (ICU) [14]. Additionally, patients with cytokine storm were more likely to experience ARDS [15] and cytokine storm is suspected to be a key player in cardiovascular injury [16]. CRP levels correlated positively with inflammatory cytokines, particularly IL-6, in COVID-19 patients and served as a biomarker for disease severity [13,15]. CRP amplified inflammatory and pro-thrombotic processes by increasing ACE expression and activity in various tissues, while ACE-produced Angiotensin II stimulated CRP production, creating an inflammation-intensifying feedback loop. CRP also upregulated Plasminogen Activator Inhibitor-1, inhibiting fibrinolysis and promoting clotting. These interactions with the ACE system and clotting cascade contributed significantly to cytokine storm pathophysiology in COVID-19, exacerbating systemic inflammation and coagulation abnormalities. Here, we will review multiple cytokines implicated in the cytokine storm phenomenon within the context of the changing cytokine landscape that occurs in aging, irrespective of COVID-19 infection, to examine the possible interaction between these elements. Additionally, we will review the use of biologics to target specific cytokines and consider further directions of such research.

## 2. Mechanisms of Cytokine Storm Induction by SARS-CoV-2 and Potential Therapeutic Targets

SARS-CoV-2 is an enveloped positive sense RNA virus that enters host cells via the angiotensin-converting enzyme 2 (ACE2) receptor via the receptor binding domain of the spike protein [17]. Once inside the cell, the virus triggers a cascade of events that results, in part, in the release of cytokines which serve as intracellular mediators that work to increase the body’s inflammatory response. However, this aberrant response also results in a cytokine storm. One proposed pathway for this derangement focuses on dual activation of NF-kappaB [18]. First, SARS-CoV-2 activates NF-κB directly via pattern recognition receptors. Simultaneously, the occupation of the ACE2 receptor by the virus, Angiotensin 2, which normally binds ACE2 and is degraded, increases in the serum and induces inflammatory cytokines such as TNF-α and IL-6 via a disintegrin and metalloprotease 17 (ADAM17). IL-6 can independently activate the signal transducer and activator of transcription 3 (STAT3), which then synergizes with NF-κB to activate the IL-6 amplifier. This amplification further enhances the activation of both NF-κB and STAT3, leading to the release of numerous cytokines and chemokines. Here we will focus on the interleukin class of cytokines, specifically IL-1, IL-6, IL-17, and IL-23, given the number of biologics already in use that target these immune mediators and offer a potential solution for preventing and treating cytokine storm.

### 2.1. IL-1

IL-1 refers to a large group of conserved proteins. IL-1α and IL-1β are the most extensively studied of the group and are collectively referred to as IL-1. IL-1 is a potent proinflammatory cytokine that plays a role in a broad range of inflammatory responses. It serves to trigger vasodilation, attract innate immunity cells to the site of tissue damage and infection, generate the fever response, and propagate hyperalgesia [19]. Specifically, IL-1 receptor activation leads to the activation of a robust downstream cascade, which includes NF-kB, that induces the expression of target genes such as IL-6, IL-8 [20].

Inflammaging is a rapidly developing field that is working to better characterize the sterile, low-grade, chronic inflammation that progressively increases with age. Studies have shown that IL-1 is elevated in the bone marrow of mice, which leads to myeloid differentiation thought to contribute to the general framework of inflammaging [21]. A study performed in mouse models found that IL-1 was a much more potent inducer of IL-6 production and secretion from adipose tissue compared to TNF-α. Furthermore, adipose tissue from aged mice produced significantly more IL-6 after stimulation by IL-1 compared to adipose tissue from young mice [22]. A study specifically on cardiac tissue from mice found that when the IL-1 receptor was knocked out, aging mice did not develop left ventricular concentric hypertrophy, a finding that was seen in wild-type mice [23]. A similar study conducted on pancreatic beta cells demonstrated that IL-1 knockout mice exhibited better preservation of beta cell function, resulting in more robust insulin secretion in response to glucose stimulation compared to controls [24]. Taken together, these studies further support the central role of IL-1 in facilitating age-related molecular and phenotypic changes in multiple organ systems.

Of the studies translating these findings into human subjects, there have been variable results. One study conducted as part of Vitality 90+, a prospective multidisciplinary population-based study of people aged 90 or older in Finland, sought to investigate the blood levels of IL-1, IL-6, and C-reactive protein (CRP) and their relationship with age. All three markers were found to be elevated in the participants who died within 4 years compared to those who did not. Additionally, even when adjusting for chronic diseases and other risk factors, IL-1 was strongly associated with mortality [25]. A recent systematic review of IL-1, IL-6, and TNF-α levels in aging individuals found only IL-6 levels to be reliably elevated in elderly patients with various disease comorbidities such as sarcopenia, stroke, and liver disease compared to disease-free controls [26].

IL-1 levels have been linked with COVID-19 infection and long-term sequelae from the SARS-CoV-2 virus. A recent investigation revealed that in a cohort of patients with mild to moderate acute COVID-19, IL-1β levels were elevated during acute infection, with concentrations surpassing those observed in patients with bacterial pneumonia. Additionally, it was seen that of the many hallmarks inflammatory mediators analyzed, only IL-1, IL-6, and TNF had a significant association with post-acute sequelae of COVID-19 such as fatigue, dyspnea, and issues with concentration [27]. Another study found that in patients with SARS-CoV-2-induced pneumonia who developed severe respiratory failure, macrophage activation syndrome was driven by IL-1 while the aspect of immune dysregulation was largely driven by IL-6 [28].

Overall, it has been established that IL-1 is essential to the primarily inflammatory response and is responsible for increasing IL-6. While a consensus has yet to be reached on the correlation of IL-1 levels in aging, it plays an important role in both aging tissue and the pathogenesis of COVID-19. Although the current thought is that IL-6 is more predictive of COVID-19 outcomes and the severity of disease progression, this finding calls for further research, especially in human subjects, to better elucidate the implication of IL-1 levels.

### 2.2. IL-6

IL-6 is a soluble mediator with a wide range of effects on immune response, inflammation, cellular development, and differentiation. Historically, during initial discovery and characterization, it was given various names based on its activity such as B-cell stimulatory factor, hepatocyte stimulating factor, hybridoma growth factor, and interferon-B2 [29]. It is now known to be associated with these functions and many more, including CD4+ T cell differentiation, osteoclast differentiation, VEGF production, and keratinocyte proliferation. Interestingly, IL-6 has also been shown to lead to permanent cellular growth arrest [30].

IL-6 is also strongly implicated in more pathological inflammatory states including the COVID-19 infection. It can serve as an early biomarker to monitor immune responses and predict disease morbidity and mortality. It has been determined that IL-6 concentrations can be elevated by over 1000 times the upper limit than normal during SARS-CoV-2 infection compared to other inflammatory mediators such as IL-1 and IL-10 which may only be elevated up to 100-fold [31]. Additionally, according to a recent study, increasing age correlated positively with serum levels of individual biomarkers including IL-6 in COVID-19 infection [32]. Elevated IL-6 levels within the context of COVID-19 infection, seen to greater extents in older individuals, can be predictive of a plethora of negative outcomes. In 2020, a research team developed the Dublin–Boston score which uses the change between two IL-6:IL-10 ratio measurements taken 4 days apart to guide clinical decision-making by identifying hospitalized patients at risk of impending poor outcomes. The score is applicable to patients both in the ICU and on the ward [33]. Another study focused on identifying the strongest predictive factors of COVID-19 mortality found that, compared with their overall cohort, mortality was significantly higher in severely ill old patients with IL-6 >/= 33 pg/mL at baseline [32]. Furthermore, a retrospective review found that maximal IL-6 level before intubation was the strongest predictive factor for the need of mechanical ventilation [34]. It is also hypothesized that IL-6, in conjunction with IL-17, may mediate vascular dysfunction and thromboembolic events [35]. From a neurological standpoint, IL-6 has been associated with depression, fatigue, and sleeping difficulties, especially in patients with long COVID [36].

IL-6 is also closely linked with physiological aging, irrespective of active infection. Recent discoveries in this area have shown that out of the serum biomarkers used to assess inflammation, C-reactive protein (CRP), and IL-6 are by far the most closely linked in pro-inflammatory clusters. Increasing levels of IL-6 correlate strongly with age, poor physical performance, and increased death rate. [37]. IL-6 is also closely linked with Plasminogen activator inhibitor-1 (PAI-1 aka SERPIN1) where elevated levels of both correlate with other clinical markers of aging such as reduced walking speed and lower grip strength [37,38].

Taken together, this suggests that COVID-19 may work synergistically with the molecular changes already present in aging to further increase IL-6 levels, which serves to propagate the morbidity and mortality of the virus.

### 2.3. IL-17

IL-17 is known to be a versatile cytokine implicated in processes such as host immune defense, tissue repair, inflammatory disease pathogenesis, and cancer progression [39]. It has been of particular interest since the discovery of the Th17 lineage of CD4+ T helper cells, a subset of CD4+ T cells that selectively express IL-17, although IL-17 may be expressed by other cell types as well [40]. The IL-17 family includes 6 members with IL-17A ubiquitously referred to as IL-17 [41]. One of IL-17’s most notable functions is its ability to induce neutrophil chemotaxis and activation, which is particularly significant since neutrophil tissue infiltration is widely regarded as a reliable indicator of acute inflammation [42]. This is particularly relevant to our discussion as the accumulation of neutrophils in the lungs following SARS-CoV-2 infection increases the release of cytokines [43]. IL-17 can increase cytokine response more directly as well by influencing the release of other inflammatory actors such as IL-1β, IL-6, and TNF-α [44].

Thus, as expected, it has been found that IL-17 levels are elevated during COVID-19 infection with higher levels in those with more severe diseases [45,46]. Similar results have been observed in studies of PBMCs with higher levels of Th17-produced IL-17 cytokines in samples taken from patients with COVID-19 ARDS [47]. Given the relationship between IL-17 and Th17 cells, it is also worth noting that in patients that progressed to critical condition there was no difference in T cell subtypes except in the percentage of Th17 cells, which was twofold higher [48]. This wider analysis aligns with an early report of increased Th17 cells in a patient with a severe immune response [49]. Attention may also be paid to the ratio between Th17 cells and Treg cells. Higher Th17 cells and IL-17 levels with decreased responses of Treg cells has been observed in patients that died of COVID-19 compared to those that improved [50].

Despite significant attention to Th17 cells and IL-17, their roles in the aging process remain unclear. Some studies report an increase in memory Th17 cells and elevated IL-17 mRNA expression with age [51], while others indicate a decline in memory Th17 cells [52], or no significant changes in IL-17 levels at all [53]. Interestingly, the study finding decreased Th17 memory cells with age also observed increased differentiation of Th17 cells from naive CD4+ cells with aging, leading to the conclusion that there was a secondary process impacting the observed decrease in memory T cells. Additionally, research suggests that increasing IL-17 levels have been linked to cognitive aging in both people and mice [54], and IL-17 is strongly implicated in skin aging in mouse models [55]. Thus, there is sufficient evidence to suggest that IL-17 plays a role in the process of growing older and that this, taken together with the notable changes that COVID-19 can inflict on the Th17 and IL-17 landscape, should be subject to our attention when considering how to best protect the vulnerable elderly population.

### 2.4. IL-23

IL-23 belongs to the IL-12 cytokine family but differs functionally and structurally from IL-12. While IL-12 (composed of the p40 and p35 subunits) promotes T helper 1 (Th1) responses, IL-23 (composed of the p40 and p19 subunits) drives the development of IL-17-producing cells and supports other mediators of chronic inflammation [56,57]. In a well-functioning immune system, IL-23 is released in response to tissue injury and insult, recruiting cells necessary for acute inflammation [58]. When out of balance, however, it has the potential to cause harm via excessive inflammatory responses and is unsurprisingly associated with several inflammatory autoimmune conditions such as psoriasis, irritable bowel disease, and rheumatoid arthritis [59,60,61].

One of the body’s first lines of defense against COVID-19 are alveolar macrophages, which are known to release IL-23 in response to lung injury [62]. Consistent with this, serum IL-23 was found to be elevated in all groups of COVID-19 patients with markedly higher values in the critical and severe groups and a significant positive correlation to CRP levels [63]. It is worth noting that there is some inconsistency in the literature regarding IL-23 levels. One study did not find significant differences in IL-23 levels in COVID-19 patients compared to healthy controls [64]; however, it also did not find significant differences in IL-17 levels, calling into question the statistical power of the study given the body of evidence outlined above in support of elevated levels of IL-17 during COVID-19 infection. The study did find that IL-23 levels were higher in male patients. This is of interest given the established higher rates of morbidity and mortality from COVID-19 in men and the differences in immune response observed by us [65,66,67]. This perhaps further suggests that IL-23 may play a role in COVID-19 severity.

With regards to IL-23 and aging, IL-23 production in mouse models increases with age and is associated with cell senescence [68,69]. However, there are limited studies evaluating IL-23 levels in the aging human population, with some evidence to suggest there may not be a correlation between IL-23 and age [70]. Despite this, what does appear clear is the potential link between IL-23 levels and several conditions common in the aging population such as atherosclerosis [71,72], diabetes [73], and hypertension [74,75]. Notably, these are also considered comorbidities for COVID-19, and therefore it is reasonable to consider IL-23 as a potential viable target for intervention when treating the most at-risk members of the elderly population for COVID-19.

## 3. Biologics Targeting IL-6, IL-1, IL-17, and IL-23

Given the significant role that cytokines play in the development and progression of COVID-19, there has been a large movement towards investigating the efficacy of biologics in this disease process. There have been a number of Phase II clinical trials, randomized controlled trials, and retrospective studies looking at the effects of tocilizumab, sarilumab, and anakinra in a variety of COVID-19 patients and sub-groups.

### 3.1. Tocilizumab and Sarilumab

Tocilizumab and Sarilumab are two biologics targeting IL-6. Tocilizumab was first approved by the FDA in 2010 for the treatment of moderate-to-severe rheumatoid arthritis. Sarilumab was more recently approved by the FDA in 2017 for Rheumatoid Arthritis treatment as well. They have both been investigated for COVID-19, with the former having been studied more extensively. As part of this study, we chose to look at Phase II trials and randomized controlled trials (RCTs) investigating Tocilizumab and Sarilumab since they are typically considered the strongest and most robust in terms of establishing causality and minimizing bias.

#### 3.1.1. Tocilizumab in Non-Critically Ill COVID-19 Patients

The RECOVERY trial, a RCT studying tocilizumab that enrolled the largest number of patients, found that in 4116 hospitalized COVID-19 patients, most of whom were already receiving steroids as part of standard of care (SOC), tocilizumab improved survival and clinical outcomes [76]. Another Phase II clinical trial involving 32 hospitalized patients with non-critical, non-intubated COVID-19 pneumonia found that low-dose tocilizumab was associated with improvement in clinical and laboratory markers of inflammation, including body temperature and CRP levels [77]. A similar Phase II trial involving 354 COVID-19 patients supported the efficacy of tocilizumab when administered early in the disease course. Notably, a sub-group analysis found that patients over the age of 79 had the greatest benefit. [78]. A RCT involving 389 hospitalized COVID-19 patients not receiving mechanical ventilation found that the tocilizumab group had a significantly lower rate of progression to mechanical ventilation or death compared to the control group [79].

#### 3.1.2. Tocilizumab in Moderate to Severe or Critically Ill COVID-19 Patients

An RCT involving 130 COVID-19 patients with moderate or severe pneumonia found that tocilizumab did not reduce the need for mechanical or noninvasive ventilation or death by day 28. Similarly, another RCT involving 438 patients with severe pneumonia showed no significant reduction in mortality at 28 days when given tocilizumab [80]. A similar RCT with 243 moderately ill patients found that tocilizumab was not effective for preventing intubation or death. Sub-group analyses further confirmed the risk factors for disease severity. Patients over the age of 65 and individuals with higher baseline IL-6 concentrations were at greater risk of progressing to worse outcomes [81]. In contrast to these results, a study conducted as part of the larger REMAP-CAP trial in 803 critically ill patients with COVID-19 found that tocilizumab and sarilumab improved survival. Of note, greater than 90% of patients were also receiving steroids as part of the SOC [82].

#### 3.1.3. Sarilumab in Non-Critically Ill COVID-19 Patients

A Phase II trial in 115 patients with hospitalized COVID-19 studied the efficacy between 200 mg versus 400 mg of sarilumab. Results showed that early IL-6 blockade with a single sarilumab dose of 400 mg was associated with better outcomes. Only 13% of patients in the sarilumab-400 group experienced disease progression compared to 25% in the sarilumab-200 group and 28% in the control cohort. [83].

#### 3.1.4. Sarilumab in Moderate to Severe or Critically Ill COVID-19 Patients

An RCT involving 416 patients with severe or critical COVID-19 found that sarilumab did not improve mortality in hospitalized patients receiving supplemental oxygen. However, results from a post-hoc analysis did show an improvement in mortality when sarilumab was administered to patients who were also receiving steroids [84]. An RCT with 1365 critically ill patients on mechanical ventilation found no improvement in outcomes with sarilumab treatment [85]. Additionally, another RCT involving 153 patients with moderate to severe COVID-19 showed no benefit from subcutaneous sarilumab when added to the standard of care regimen [86].

### 3.2. Anakinra

Anakinra is an IL-1 receptor antagonist originally approved in 2005 by the FDA for moderate-to-severe active rheumatoid arthritis (RA) in adult patients who failed one or more disease-modifying antirheumatic drugs (DMARDs). During the COVID-19 pandemic, it was investigated for its efficacy in this disease.

#### 3.2.1. Anakinra in Non-Critically Ill COVID-19 Patients

A RCT of 30 patients found that anakinra reduced the need for invasive mechanical ventilation and reduced hospital length of stay [87]. An RCT of 119 patients with mild-to-moderate COVID-19 pneumonia found that anakinra did not improve outcomes [88]. The SAVE-MORE RCT evaluated the efficacy and safety of anakinra, an IL-1α/β inhibitor, in 594 patients with COVID-19 at risk of progressing to respiratory failure (identified by plasma soluble urokinase plasminogen activator receptor serum levels). This study demonstrated the efficacy of anakinra, an IL-1α/β inhibitor, in patients with COVID-19 and high serum levels of soluble plasminogen activator receptor [89]. A similar RCT of 130 patients found that administration of anakinra, guided by suPAR levels, decreased the incidence of systemic respiratory failure [90].

#### 3.2.2. Anakinra in Moderate to Severe or Critically Ill COVID-19 Patients

An RCT involving 71 patients with deteriorating COVID-19 respiratory symptoms found that not only was anakinra not effective but was also inferior to optimized SOC [91]. Another RCT of 195 severe COVID-19 patients with a high risk for deterioration was studying the efficacy of tocilizumab and anakinra. Although it ended early because tocilizumab became standard of care, analysis found that anakinra or tocilizumab did not significantly shorten the time to clinical recovery compared to usual care [92]. Additionally, a large RCT done in 342 COVID-19 patients with signs of cytokine release syndrome found that drugs targeting IL-1 (anakinra) or IL-6 (tocilizumab or siltuximab) did not shorten the time to clinical improvement [93]. An RCT with 179 patients with severe COVID-19 found that anakinra did not prevent the need for mechanical ventilation or reduce mortality risk compared with the SOC [94].

Overall, the literature presents mixed findings on the efficacy of tocilizumab, sarilumab, and anakinra. However, evidence suggests that these biologics are most effective when administered early in the disease course to patients without severe pneumonia and those who are not critically ill [95]. There are also results suggesting that concomitant corticosteroid administration augments the efficacy of biologics.

### 3.3. Secukinumab and Ixekizumab

Secukinumab and ixekizumab are both monoclonal antibodies that target IL-17. To our knowledge, no randomized controlled trials exist that test secukinumab as treatment for COVID-19 and only one exists for ixekizumab. However, there is a growing body of research supporting that both antibodies may be safe for use in COVID-19 and a small emerging body of evidence that supports possible benefits from secukinumab. Given the limited research on these monoclonal antibodies, research could not be broken down by disease severity.

At the start of the pandemic, multiple case reports on secukinumab and COVID-19 were published. One of the first reports came out of Italy in March of 2020 where a 73-year-old woman with psoriasis, psoriatic arthritis, and hypertension was receiving treatment with secukinumab at the time she contracted COVID-19 [96]. Her infection resulted in only mild symptoms despite the risk factors of her age and comorbid condition of hypertension. Shortly after, this case was discussed alongside seven others, one novel and six from the existing literature [97]. All patients were being treated with secukinumab for psoriatic arthritis, psoriasis, or ankylosing spondylitis. Three of the patients did not require hospitalization and of the other four, three made a full recovery and were discharged home. The final of the seven required mechanical ventilation. There was additionally an early case report that looked at a cohort of 151 patients who had previously been studied for a 136-week trial of treatment with secukinumab [98]. Of these patients, 119 were still taking secukinumab, and none of these 119 patients had contracted COVID-19 by May of 2020 when the report was submitted. In addition to case reports, in May of 2021, an open-label comparative study that compared the use of secukinumab and baricitinib, an inhibitor of Janus Kinase, to the use of baricitinib alone, found that the secukinumab group required significantly less ICU support and intubation and additionally saw improved 30-day all-cause mortality [99].

In terms of ixekizumab, one of the original reports of a patient taking the drug and contracting COVID-19 occurred in April of 2020. The patient was a 46-year-old male with type I Brugada syndrome, arterial hypertension, and plaque psoriasis [100]. The patient developed COVID-19 symptoms one week after his first dose of ixekizumab and eventually presented it to the hospital where he was admitted in part due to low oxygen saturation. He was discharged after 22 days. Since he had only received one dose of the regimen, the authors chose not to speculate on its role in impacting COVID-19 infection. Around the same time, a case report was published on a 55-year-old man who was completing his induction regimen with ixekizumab when he tested positive for COVID-19 and was ultimately asymptomatic [101].

Since the start of the pandemic, numerous larger-scale trials have been published to look at IL-17 inhibition in COVID-19. One systematic review looked at nine observational studies of 7106 patients with psoriasis using either IL-17 inhibitors or non-biologics [102]. The study found no significant differences in the rates of infection, hospitalization, or mortality. However, a retrospective cohort study assessing 190 patients with either psoriatic arthritis or ankylosing spondylitis being treated with secukinumab found that the duration of fever was significantly shorter, and that cough and sputum were significantly reduced in the secukinumab group compared to the control group [103]. No conclusions could be drawn about hospitalization since only 5 of the total participants required hospitalization. Finally, the only randomized controlled trial to exist for IL-17 inhibition as treatment for COVID-19 looked at the use of ixekizumab, low-dose IL-2, or colchicine (an indirect IL-6 inhibitor) compared to standard of care in 60 participants [104]. The study found no significant differences in clinical outcomes. The authors did note trends in cytokine profiles in the treatment groups with decreasing pro-inflammatory cytokines and increasing anti-inflammatory cytokines, but ultimately none were statistically significant.

### 3.4. Risankizumab, Ustekinumab, and Guselkumab

IL-23 is another cytokine that can be targeted by existing monoclonal antibodies therapies. Risankizumab, ustekinumab, and guselkumab all target different subunits of IL-23, with ustekinumab notably targeting both IL-12 and IL-23. IL-23 inhibition, similar to IL-17, is largely lacking in randomized controlled trials with the current research mainly being composed of case studies.

A growing body of case studies suggests that IL-23 inhibition in patients with COVID-19 does not worsen clinical outcomes. One case study on risankizumab in a 37-year-old patient with psoriasis reported that the patient made a full recovery and was able to continue with his dosing schedule without issues [105]. Another case study in a 32-year-old patient with psoriasis and psoriatic arthritis on guselkumab also achieved full recovery from COVID-19 [106], and yet another patient in their 40s with psoriasis on ustekinumab required no hospitalization or medications other than over-the-counter acetaminophen for fever reduction to make a full recovery [107]. Additional reports highlight cases such as a 44-year-old woman with Crohn’s disease on ustekinumab, who made a full recovery [108], and a 40-year-old man with plaque psoriasis on risankizumab, who contracted COVID-19 ten days after his injection but experienced only three days of symptoms—significantly shorter than the average of eight days in the general population [109].

Given the ages and lack of significant risk factors for severe COVID-19 infection, these cases are perhaps less interesting than the report of a 77-year-old male smoker with psoriasis on risankizumab, along with an additional medical history including hypertension, previous myocardial infarction, and chronic obstructive pulmonary disease. The patient required hospitalization for COVID-19 for three weeks but was ultimately discharged from the hospital in good clinical condition and was reported to be free of complaints on follow up [110]. Even more compelling is the report of a 40-year-old woman suffering from psoriasis who contracted COVID-19 and self-administered her guselkumab injection while actively infected. The day after the injection, she reported a rapid improvement in her shortness of breath, fevers, myalgia, and fatigue [66].

Although no randomized controlled trials exist to investigate IL-23 inhibition in COVID-19 infection, there is one cross-sectional cohort study that can bolster the observations made in the case studies. The cross-sectional cohort study looked at 732 patients with psoriasis and found that IL-23 inhibitor use was associated with a significantly decreased risk of COVID-19 compared to patients using topical ointment, methotrexate, narrowband ultraviolet therapy (NB-UVB), TNF-α inhibitors, IL-17 inhibitors, and traditional Chinese medicine [111]. Additionally, patients using IL-23 inhibitors were more likely to be asymptomatic after recovery compared to patients using methotrexate, NB-UVB, or TNF-α inhibitors.

In sum, the existing body of literature on IL-23 inhibition in COVID-19 infection, although not yet robust, certainly encourages further exploration.

## 4. Conclusions

COVID-19 presented a unique challenge to the medical world. Research advanced at an unprecedented rate and included multiple important discoveries that helped guide vaccine and therapeutic development. One valuable piece of knowledge gained was the role of the cytokine storm. It became evident that grossly elevated numbers of cytokines were contributing to the severity of disease. As time passed, it also became clear that the elderly were disproportionately impacted by the virus with higher rates of morbidity and mortality. This prompted us to further investigate the links between cytokine levels and age.

We found that the elderly at baseline have elevated levels of multiple cytokines that are implicated in cytokine storm, a concept included in the theory of inflammaging [112]. This may predispose the aging population to cytokine storm during COVID-19 infection, with clear evidence supporting the link between IL-6 levels and disease severity, with moderate evidence of contribution from other cytokines, including IL-1, IL-17, and IL-23, discussed at length above.

Given these findings, we explored the current research on the use of biologics targeting specific cytokines known to play a role in the cytokine storm phenomenon. The strongest research conducted involved the use of IL-6 inhibitors (Tocilizumab and Sarilumab). These studies were often conducted with specific groups of patients, such as those that were critically ill. This helped elucidate the optimal timing of drug administration as well as the patient population that benefited the most. Overall, it was found that Tociliuzmab was most effective when administered early in the disease course to patients with COVID-19 that were not critically ill.

Although data for IL-6-targeting biologics included multiple gold standard randomized controlled trials, there is a lack of high-quality studies for biologics targeting other cytokines. Therefore, concepts such as the timing of optimal drug administration have yet to be studied for the other biologics. Additionally, based on the findings of Hasan et al., 2021 [99], which found a benefit in using both a Janus Kinase inhibitor and an IL-17 inhibitor, there may be reason to investigate the use of multiple immune modulators in achieving optimal impact.

Finally, it is noteworthy that, to our knowledge, none of the published studies specifically target the elderly population, despite their increased vulnerability to severe COVID-19 outcomes. This research gap underscores the importance of developing more targeted and effective treatments for elderly patients and emphasizes the need to consider age-related factors in both research design and clinical practice. As we continue to face global health challenges, including potential future pandemics, these insights will be crucial in shaping our approach to disease management, public health strategies, and healthcare policy.

## Data Availability

No new data were created or analyzed in this study. Data sharing is not applicable to this article.
